# Descriptive study on substance uses and risk of sexually transmitted infections in the practice of Chemsex in Spain

**DOI:** 10.3389/fpubh.2024.1391390

**Published:** 2024-05-10

**Authors:** Pablo del Pozo-Herce, Enrique Baca-García, Antonio Martínez-Sabater, Elena Chover-Sierra, Vicente Gea-Caballero, Javier Curto-Ramos, Michal Czapla, Piotr Karniej, Jesús Martínez-Tofe, Mercedes Sánchez-Barba, Regina Ruiz de Viñaspre, Raúl Juárez-Vela

**Affiliations:** ^1^Department of Psychiatry, Fundación Jimenez Diaz University Hospital, Madrid, Spain; ^2^Instituto de Investigación Sanitaria de la Fundación Jiménez Díaz, Madrid, Spain; ^3^Nursing Department, Nursing Care and Education Research Group (GRIECE), Universitat de Valencia, Valencia, Spain; ^4^Care Research Group (INCLIVA), Hospital Clinico Universitario de Valencia, Valencia, Spain; ^5^Internal Medicine, Consorci Hospital University of Valencia, Valencia, Spain; ^6^Faculty of Health Sciences, Research Group Community Health and Care, International University of Valencia, Valencia, Spain; ^7^Department of Psychiatry, Clinical Psychology and Mental Health, La Paz University Hospital, Madrid, Spain; ^8^Department of Emergency Medical Service, Faculty of Health Sciences, Wroclaw Medical University, Wrocław, Poland; ^9^Department of Nursing, Faculty of Health Sciences, Research Group in Care, University of La Rioja, Logroño, Spain; ^10^Faculty of Finance and Management, WSB University in Wroclaw, Wroclaw, Poland; ^11^Department of Statistics, Faculty of Medicine, University of Salamanca, Salamanca, Spain; ^12^Faculty of Medicine, Biomedical Institute of Salamanca, Prevention and Early Intervention in Mental Health, University of Salamanca, Salamanca, Spain

**Keywords:** substance use, HIV infections, sexual behavior, STIs, pre-exposure prophylaxis, sexually transmitted infection

## Abstract

**Background:**

In recent years, there has been an increasing use of sex-related substances (known as “Chemsex”) to facilitate, intensify, and prolong the sexual experience of men who have sex with men. This phenomenon poses a public health problem, increasing the risk of sexually transmitted infections (STIs) and mental disorders.

**Objective:**

The primary aim of this study was to delve into the correlation between substance use and sexual health, specifically examining the association between different substances used and the risk of sexually transmitted infections (STIs) in the context of Chemsex in Spain.

**Methods:**

An observational, descriptive, cross-sectional study was conducted among 563 Spanish participants between January and April 2023. Non-probabilistic purposive sampling was used by the investigators. The researchers administered a questionnaire to men who have sex with men who use substances, especially in the sexual sphere, in all the autonomous communities of Spain.

**Results:**

14.7% reported having practiced slamsex in the last year, and 17.94% were diagnosed with a Sexually Transmitted Infection in the previous 6 months. Of these, 21% were on PREP treatment, with the main STIs being gonorrhea (*p* < 0.001), chlamydia (*p* < 0.001), genital herpes (*p* = 0.020), and syphilis (*p* < 0.001). The 63.7% used methamphetamines as the main drug in the practice of chemsex.

**Discussion:**

Chemsex in Spain is linked to a high prevalence of STIs, especially gonorrhea and chlamydia, even among those on PrEP treatment. The use of various drugs during chemsex, such as amyl nitrite, GHB, ecstasy, and others, correlates with higher rates of STIs, highlighting the need for interventions to reduce risk and harm. The drugs most associated with slamsex include ketamine, mephedrone, and methamphetamine, underscoring the importance of addressing the risk behaviors associated with this activity.

**Conclusion:**

This study shows that chemsex appears to be associated with a high prevalence among men who have sex with men. Who use multiple substances in a sexual context, and are particularly exposed to sexually transmitted infections (STIs), indicating a particular need for STI prevention and care in this group.

## 1 Introduction

In recent years, there has been an increase in the intentional use of drugs for sex, which can last for hours or even days, among men who have sex with men (MSM) (1–6). The recreational uses of substances vary in place or environment. Some of these recreational consumptions take place in sexual environments, a term known in Europe as sexualized substance use, where we include “Chemsex” linked to the LGTBIQ+ sexual culture (7). However, we find other denominations in the world, such as “*Intensive sex partying*” in Australia (8), “*Party and Play*” (*PnP*) in North America, or even “*sexualized drugs*” used more in the general population (9). This shows that it is a global problem present at the international level.

The Chemsex phenomenon has been linked to risky sexual behaviors, as well as an increase in sexually transmitted infections (1, 10–13). According to the latest European online survey for MSM (EMIS - 2017), the section on substance use revealed the prevalence of substance use for sexual purposes in the last 12 months was 14.1% (14, 15), with Spain being one of the countries with the highest prevalence of chemsex at the European level (16, 17). The substances most commonly used in the practice of Chemsex include methamphetamine, mephedrone, and other synthetic cathinones, gamma-hydroxybutyric acid/gamma-butyrolactone (GHB/GBL) (1, 2, 9, 14, 15, 18–26) alkyl nitrites (poppers) (27) medication for erectile dysfunction (6, 28–30) and less frequently other substances such as cocaine and ketamine (13, 22, 31). If the consumption of these psychoactive substances is intravenous (19, 32) in this context, it is called slamming or slamsex (1, 6, 14, 25, 28, 29).

Several sexual practices have been described in context of chemsex, such as unprotected anal intercourse or “*bareback*” (33, 34) “*fist-fucking”* (19, 32, 33, 35) group sex (33) which have been associated with an increased risk of being diagnosed with sexually transmitted infections (*STIs*) such as gonorrhea, chlamydia, syphilis (6, 11, 18, 21, 23–27, 29, 32, 35–38), hepatitis C (*HCV*) and HIV (15, 19, 22, 30, 39). For this reason, chemsex is considered a health concern due to the increase in HIV primary infection, hepatitis C reinfections (14), and, in general, a higher probability of acquiring sexually transmitted diseases (*STDs*) (1, 14, 23). A higher incidence has been observed in PLHIV (People living with HIV) versus MSM who do not have HIV (15, 25). As a consequence, PLHIV are at increased risk for mental health problems (6, 25, 32, 34), specifically anxiety and depression (23, 36). In the same vein, Chemsex practices have been associated with increased risk of psychosis (6, 14) and delusional thoughts due to chronic methamphetamine use (29, 40), suicidal ideation (6, 41) anxiety, depression (14, 34), overdose (15), behavioral disturbances (6), substance addiction (26) and reduced adherence to PrEP treatment (6).

The reasons why MSM engage in chemsex can be diverse, including increasing libido (27), intensifying sexual pleasure (18), facilitating sexual euphoria and disinhibition (39), better management of negative emotions such as lack of self-confidence during sex (14,40.43), improved perceived quality of sex and relationship with internalized homophobia (14), prolonged duration of sex sessions (1) and realization of sexual fantasies.

Furthermore, the rapid changes in substance consumption, including the expansion of new psychoactive substances (NPS) reported in Europe (42), underscore the need to explore the profile of substance consumption in sexual contexts among MSM. Understanding these profiles could provide insights into the possible connection between chemsex and MSM participation in these practices, particularly in light of the association between chemsex and sexually transmitted infections (STIs), as well as the emergence of antibiotic-resistant infections.

## 2 Materials and methods

### 2.1 Study design

An observational and descriptive cross-sectional study was conducted between January and April 2023.

### 2.2 Population and scope of the study

The study was conducted across all autonomous communities of Spain. The investigators employed non-probabilistic purposive sampling. The sample consisted of n = 563 participants who met the following inclusion criteria: (1) being over 18 years old, (2) identifying as male, (3) experiencing attraction to men or identifying as MSM (men who have sex with men), (4) possessing proficiency in Spanish to complete the questionnaire, and (5) engaging in both sexual relations and substance use. Exclusion criteria included: (1) refusal to provide informed consent and (2) participants who incorrectly filled out or left the questionnaire incomplete.

### 2.3 Sociodemographic characteristics

Participants were asked to provide a series of sociodemographic characteristics as part of the survey data related to the study phenomenon. These characteristics included age, gender identity, sexual behavior, marital status, autonomous community, employment status, socioeconomic level, monthly income, level of education, and relationship status.

### 2.4 Study variables

To investigate substance use, participants were asked whether, within the past 12 months, they had utilized substances both in a sexual context to enhance, heighten, or extend sexual activity and in a non-sexual context.

The substances included in the questionnaire were amyl nitrites, medication for erectile dysfunction GHB/GBL, alcohol, tobacco, ecstasy, amphetamines, ketamine, methamphetamine, mephedrone, cannabis, cocaine, opioid analgesics, and heroin. The use of more than one of these substances in connection with the practice of Chemsex was considered poly-drug use. Participants were also asked about administration routes (oral or injecting). A new category called “Other substances” was introduced, allowing participants to specify any substance they used that wasn’t listed among the provided options.

The questionnaire featured a comprehensive list of substances, including tobacco, alcohol, cannabis, and cocaine. These substances were explicitly included due to their frequent association with the practice of chemsex, as supported by existing literature. Moreover, other substances commonly linked to chemsex were also included in the questionnaire, ensuring a thorough examination of substance use patterns in this context (39).

The survey was created digitally using the Microsoft Forms® platform and was subsequently migrated to the www.estudioenfermeria.com platform for digital dissemination and intellectual property control to increase methodological control and avoid duplication. The online questionnaire received 1,179 responses. Participants were recruited through disseminating the questionnaire on social networks, LGTBI-related websites, social media channels, sexual health clinics, geolocation applications, and LGTBI groups.

Participants were not offered any incentives for taking part in the study. All data were collected voluntarily, anonymously, and confidentially. The questionnaire typically required around 10 to 20 min to complete. Participants were provided with the principal investigator’s contact email address for any queries regarding the questionnaire or the study. They were explicitly informed that completing the questionnaire was voluntary and their participation was anonymous and confidential. They were also assured that they could withdraw from the study at any point if they wished to do so.

### 2.5 Risky sexual practices and STIs testing

Participants were queried about their marital status and if they were in a committed relationship, as well as whether they were currently using pre-exposure prophylaxis (PrEP). Moreover, participants were prompted to disclose if they had contracted any sexually transmitted infections (STIs) within the past 6 months. The STIs listed in the questionnaire encompassed Gonorrhea, Chlamydia, Genital Herpes, Syphilis, HIV, Monkeypox, and Hepatitis. A category “other STIs” was added, in which participants could specify whether they had acquired any STIs not previously collected in sexual practice in the last 6 months. A category was also collected where they had not had any STIs in the previous 6 months. The reason for choosing 6 months is because there has been an increase in STIs in the last few months (38, 43).

### 2.6 Statistical analysis

Descriptive analyses were conducted to examine the sociodemographic characteristics of the sample. Frequencies of Chemsex behaviors and other risky sexual practices were computed. Furthermore, differences between homosexual and heterosexual men concerning substance use during Chemsex were investigated. The chi-square statistic was used for STI diagnosis and use of pre-exposure prophylaxis.

The dataset underwent systematic cleaning, which included addressing missing values and identifying outliers. Demographic characteristics (such as age, gender, etc.) and substance use variables (e.g., alcohol, stimulants, etc.) were selected for analysis. Differences between individuals reporting Chemsex and those who did not were examined using the *χ*^2^ test for categorical data, with significance set at *p* < 0.05.

Additionally, participants engaging in Chemsex were stratified based on self-reported injection drug use in the previous 12 months. Differences between these subgroups were analyzed using the same statistical tests employed for Chemsex use. Logistic regression was then utilized to explore factors associated with Chemsex use, with independent variables including demographic characteristics, substance use patterns, and injection drug use status.

## 3 Results

### 3.1 Descriptive results

The final sample consisted of 563 men who have sex with men and reported substance use, residing in Spain, with ages ranging from 18 to 55 years (mean age = 36, SD = 9.20). Among the participants, 66.1% identified as gay (*n* = 372). Approximately half of the participants (50%, *n* = 264) reported completing university studies. The majority (81.3%) were employed steadily on a full-time basis. About half of the participants (49%, *n* = 276) reported being single. Approximately half of the sample (47.3%) reported having a socioeconomic level above 2,000 euros per month (see Table 1).

**Table 1:** Sociodemographic characteristics (*n* = 563).

Variables	*N*	%
Age (mean, SD)	36.91	SD 9.20
Gender identity		
Cis	550	97.7
Trans	2	0.3
Queer	11	2
Sexual identity		
Gay/homosexual	372	66.1
Heterosexual	134	23.8
Bisexual	57	10.1
Spanish autonomous community		
Andalusia	29	5.2
Aragon	21	3.7
Principality of Asturias	8	1.4
Balearic Islands	8	1.4
Canary Islands	11	2
Cantabria	10	1.8
Castilla y Leon	26	4.6
Castilla – La Mancha	16	2.8
Catalonia	49	8.7
Valencian community	52	9.2
Extremadura	6	1.1
Galicia	14	2.5
Madrid	249	44.2
Murcia Region	5	0.9
Navarre	6	1.1
Basque country	20	3.6
La Rioja	32	5.7
Ceuta	0	0
Melilla	1	0.2
Relationship status		
Single	276	49
Single with a domestic partner	166	29.5
Married	100	17.8
Divorced	9	1.6
Widower	1	0.2
Others	11	2
Employment status		
Full-time employed	458	81.3
Part-time employed	42	7.5
Unemployed	27	4.8
Retired	4	0.7
Others	32	5.7
Monthly net income		
< 499 Euros	14	2.5
500–999 Euros	19	3.4
1.000–1.499 Euros	99	17.6
1.500–1.999 Euros	165	29.3
2.000–2.499 Euros	127	22.6
2.500–2.999 Euros	67	11.9
3.000–4.999 Euros	61	10.8
more than 5.000 Euros	11	2
Educational level		
Primary school	14	2.5
Baccalaureate	39	6.9
Diploma/Bachelor’s degree	264	46.9
Master’s degree	213	37.8
PhD	33	5.9

### 3.2 Sexual risk behaviors, sexually transmitted infections, and PREP among chemsex MSM

When participants were queried about whether they had received a diagnosis of any sexually transmitted infection (STI) within the past 6 months, 101 participants (17.94%) responded affirmatively. Twenty-one percent of the sample was on PrEP treatment. Regarding participants on PrEP treatment, 35 (29.1%) were diagnosed with gonorrhea, 28 (23.3%) with chlamydia, 10 (8.33%) with genital herpes, and 21 (17.5%) with syphilis. As for participants who were not on PrEP treatment, 33 (7.44%) were diagnosed with gonorrhea, 25 (5.64%) with chlamydia, 15 (3.38%) with genital herpes, and 24 (5.41%) with syphilis. A statistically significant association was found between PrEP use and sexually transmitted infections: gonorrhea (*p* < 0.001); chlamydia (*p* < 0.001); genital herpes (*p* = 0.020) and syphilis (*p* < 0.001); no significant differences were found with HIV, Monkeypox, hepatitis, and other syphilis. The following table shows the relationship between taking PREP and having STIs (see Table 2).

**Table 2:** Relationship between take of PrEP and STIs (*n* = 563).

	Yes PrEP		No PrEP		
Have you had any STIs acquired in the last six months?	*N* = 120	%	*N* = 443	%	*p*-value
Gonorrhea	35	29.16%	33	7.44%	0.000
Chlamydia	28	23.33%	25	5.64%	0.000
Genital herpes	10	8.33%	15	3.38%	0.020
Syphilis	21	17.5%	24	5.41%	0.000
HIV	3	2.5%	14	3.16%	0.708
Monkeypox	2	1.66%	8	1.80%	0.918
Hepatitis	2	1.66%	6	1.35%	0.798

STIs, Sexually Transmitted Infection; HIV, Human Immunodeficiency Virus; PrEP, Pre-exposure Prophylaxis.

### 3.3 Sexual risk behaviors and risky substance use among chemsex MSM

63.7% of the participants in the sample used methamphetamine as their primary chemsex drug, followed by 61.6% who used mephedrone, 61.2% amphetamines, 60.3% medication for erectile dysfunction and 60.2% GHB/GBL. Table 3 shows the distribution of the participants according to the primary drug of use. Statistically significant differences were found for substances consumed and STIs amyl nitrile (*p* = 0.000); medication for erectile dysfunction (*p* = 0.000); GHB/GBL (*p* = 0.000); Alcohol (*p* = 0. 000); Tobacco (*p* = 0.001); Ecstasy (*p* = 0.000); Amphetamines (*p* = 0.000); Ketamine (*p* = 0.000); Methamphetamine (*p* = 0.000); Mephedrone (*p* = 0.000); Cocaine (*p* = 0.000); opioid analgesics (*p* = 0.014) respectively. For example, 39.8% of GHB/GBL users had STIs vs. 18.93% of non-users, so we found statistically significant differences between users and non-users vs. GHB (*p* = 0.000). No statistically significant differences were found between THC and Heroin consumption with having STIs (*p* = 0.138; *p* = 0.512) respectively.

**Table 3:** Prevalence of substance use among sexually transmitted infections (STIs) men (*n* = 563).

Substance use 12 months in a sexual context		ITS		No ITS		
Variables		*N*	%	*N*	%	*P*-value
Amyl nitrite (Poppers) (*n* = 260)	Yes	132	50.7%	128	49.2%	0.000
	No	41	13.5%	262	86.5%	
Medication for erectile dysfunction (*n* = 111)	Yes	44	39.7%	67	60.3%	0.000
	No	102	22.5%	350	77.5%	
GHB/GBL (*n* = 151)	Yes	60	39.8%	91	60.2%	0.000
	No	78	18.93%	334	81.07%	
Alcohol (*n* = 264)	Yes	157	59.5%	107	40.5%	0.000
	No	62	20.7%	237	79.3%	
Tobacco (*n* = 161)	Yes	96	50.7%	65	40.3%	0.001
	No	104	25.9%	298	74.1%	
Ecstasy (*n* = 67)	Yes	33	49.3%	34	50.7%	0.000
	No	135	27.2%	361	72.8%	
Amphetamines (*n* = 49)	Yes	19	38.8%	30	61.2%	0.000
	No	139	27%	375	73%	
Ketamine (*n* = 48)	Yes	23	48%	25	52%	0.000
	No	144	28%	371	72%	
Methamphetamine (*n* = 58)	Yes	21	36.3%	37	63.7%	0.000
	No	132	26.1%	373	73.9%	
Mephedrone (*n* = 133)	Yes	51	38.4%	82	61.6%	0.000
	No	87	20.2%	343	79.8%	
THC (*n* = 66)	Yes	41	62.2%	25	37.8%	0.138
	No	144	29%	353	71%	
Cocaine (*n* = 114)	Yes	59	51.7%	55	48.3%	0.000
	No	114	25.4%	335	74.6%	
Opioid Analgesics (*n* = 11)	Yes	4	36.4%	7	63.6%	0.014
	No	162	29.3%	390	70.7%	
Heroin (*n* = 1)	Yes	1	100%	0	0%	0.512
	No	169	30%	393	70%	

### 3.4 Sexual risk behaviors and injecting substance use (slamsex) among chemsex MSM

14.7% (*n* = 83) of the participants practiced slamsex in the last 12 months. The most commonly used injecting substances were mephedrone (*n* = 53), methamphetamine (*n* = 21), ketamine (*n* = 8), and to a lesser extent heroin (*n* = 1). Table 4 illustrates a statistically significant association between injecting substance use (slamsex) and the diagnosis of sexually transmitted infections (STIs).

**Table 4:** Prevalence of injecting substance use among men with sexually transmitted infections (STIs).

Substances injected in a sexual context for 12 months (*n* = 83)	ITS			*P*-value
Ketamine (*n* = 8)	Yes	6	75%	0.005
	No	2	25%	
Methamphetamine (*n* = 21)	Yes	12	57.1%	0.006
	No	9	42.9%	
Mephedrone (*n* = 53)	Yes	35	65.4%	0.000
	No	18	34.6%	
Heroin (*n* = 1)	Yes	1	100%	0.126
	No	0	0%	

## 4 Discussion

Our research aimed to explore the profile of men who have sex with men in a sexual context since it could explain the relationship between chemsex, sexual behavior, and the increase in the diagnosis of STIs. The profile of users who practice chemsex is that of a man between 18 and 55 years old, resident in Spain, with university studies and a permanent job. Previous research has indicated that individuals aged between 36 and 45 years old are more inclined to engage in chemsex compared to other age groups (44). These findings align with our study, where the average age of substance use and participation in chemsex was 36 years old.

Our research concludes that being a young man, living in large cities, and being exposed to environmental stressors and substance use in the last 12 months may be additional risk factors associated with a high prevalence of chemsex. These factors are in line with other studies that have scientifically established that the homosexual population is potentially up to twice as prone to experiencing psychotic symptoms compared to the heterosexual population (45, 46). Chemsex has been identified as a coping mechanism for the stressors that men who have sex with men (MSM) encounter regularly (45, 47).

The research presents an alarming picture of the prevalence of sexually transmitted infections (STIs) among men who have sex with men (MSM) involved in chemsex and slamsex. According to the study data, 17.94% (*n* = 101) of the participants reported being diagnosed with any STI in the last 6 months, data that is consistent with other studies (15, 30). An important finding of our research and worth noting is that 21% of the sample was on treatment with pre-exposure prophylaxis (PrEP), observing a strong association between being on treatment with PrEP and the diagnosis of gonorrhea, chlamydia, genital herpes, and syphilis, which also confirms the results of similar studies (35, 48).

This is striking, as one might expect those on PrEP to be less exposed to STIs due to their greater awareness of sexual health and regular intake of preventive medication. However, the results show that, among participants on PrEP, there were higher rates of diagnosed STIs: 29.16% for gonorrhea, 23.33% for chlamydia, 8.33% for genital herpes, and 17.5% for syphilis. In comparison, those who were not on PrEP also had a significant prevalence of STIs, but to a lesser extent: 7.44% for gonorrhea, 5.64% for chlamydia, 3.38% for genital herpes, and 5.41% for syphilis. Thus, there is an increasing trend among MSM in treatment with PrEP who practice chemsex with higher rates of STI diagnoses (18, 29, 32, 35–37, 48–50). For all of the above, harm reduction strategies and community-based chemsex approaches that combine support services with health promotion and education activities should be pursued (51–53). Given the entry of PrEP into the service portfolio of the National Health System (SNS), the Secretariat of the National AIDS Plan (SPNS) launched the SIPrEP in 2020, an information and monitoring system for the implementation. Running and implementation in the different Autonomous Communities (CCAA) of the PrEP programs, with the aim of collecting information on the number of participants, their characteristics, their clinical evolution and the effectiveness of PrEP. Thus, up to and including 2023, the estimated number of users who were taking PrEP is 18,075 people (54).

On the other hand, substance use appears to be associated with an increased risk of STIs in general. MSM who engage in chemsex have been reported to be at increased risk of transmitting STIs and at increased risk of engaging in risky sexual behaviors, and acquiring HIV (55). People who use amyl nitrite, GHB/GBL, alcohol, ecstasy, amphetamines, ketamine, methamphetamine, mephedrone, and cocaine have a higher proportion of STIs compared to those who do not use these substances. When considering substance use in the context of chemsex and slamsex, the results show a significant association between the use of certain substances and the prevalence of STIs. For example, 39.8% of GHB/GBL users had STIs compared to 18.93% of non-users, suggesting that the use of these substances is associated with an increased risk of STIs. Data that are also consistent with other studies in which drugs used in the chemsex context were related to STIs and sexual risk behaviors (30, 56–61). Moreover, the findings of a previous study demonstrated a 2.83-fold increase in the likelihood of acquiring a sexually transmitted infection (STI) among chemsex users (30). This result aligns with the outcomes of the current study.

These data reinforce the need for continued sexual health education and safer practices, even among those on PrEP. The high prevalence of STIs among participants on PrEP suggests that access to preventive medication is not sufficient to eliminate STI risk and that other factors, such as sexual risk behavior, may be at play.

In regards to slamsex, it is notable that a significant percentage of participants in our study reported consuming injected substances (14.7%) in the past year compared to those who did not inject drugs. Among individuals who engaged in slamsex, a higher frequency of STI diagnoses was observed, consistent with findings from other studies. For instance, research has shown that 15.7% of individuals who practiced slamsex in the previous year reported a higher frequency of STI diagnoses and engaged in high-risk sexual behaviors (29). Other studies focused on drug use and STI risk have shown an increased risk of STI acquisition by people who use drugs (39), which may be related to the search for new sensations and experiences as well as disinhibition. Risk behaviors associated with chemsex, such as having multiple sexual partners, having unprotected sex, engaging in fisting and slamsex, as well as sharing injection equipment, may be risk factors for acquiring STIs in the past 6 months in the context of chemsex (29, 36, 45).

Along the same lines, the most commonly consumed injecting drugs during slamsex associated with STI diagnosis in our study were ketamine (75%), followed by mephedrone (65.4%), and methamphetamine (57.1%). The potent stimulant effect of mephedrone has been associated with a heightened risk of engaging in high-risk sexual behaviors, which in turn increases the risk of HIV infection and other sexually transmitted infections (STIs) (62). Additionally, mephedrone use has been linked to severe psychiatric symptoms, intoxications, and dependence on injecting drugs (29). This includes a range of adverse effects such as binge drinking, anxiety, binge eating, withdrawal symptoms, and psychotic symptomatology resulting from injecting mephedrone use (6, 29, 63).

The significant prevalence of substance use within the context of chemsex, coupled with its correlation with risky sexual behaviors, underscores the importance of targeting men who have sex with men (MSM) as a critical population for prevention strategies. These findings support the need for comprehensive interventions that address both substance use and sexual health to reduce STI risk in vulnerable populations, especially in populations where chemsex is prevalent, as appears to be the case in Spain and who are on PrEP treatment. Addressing chemsex can yield broader positive impacts on individuals’ mental, sexual, social, financial, and overall health. A retrospective case review conducted in a London service found that half of MSM who reported engaging in chemsex perceived subsequent adverse physical and mental health consequences, highlighting the need for comprehensive support and intervention strategies (32, 64).

Furthermore, there exists an association between engaging in chemsex and the heightened risk of experiencing emotional instability, including symptoms of anxiety and depression. These findings align with numerous previous studies conducted among MSM, which have consistently reported a link between drug use for sexual purposes and an increased likelihood of experiencing depressive outcomes (39). Indeed, some studies have identified anxiety as a predictor in the relationship between psychosis and chemsex (29). Conversely, other research has found no significant differences in anxiety levels, although it did reveal that chemsex users exhibited higher scores on the GAD-7 scale (34). This may suggest that the use of substances to extend time in a sexual context may provoke depressive symptoms with ideas of guilt and ruin of the people who practice it, or also, it may be depressive symptoms such as social isolation and loneliness that lead to substance use in a sexual context to disinhibit themselves and be a way to socialize with other people. Our findings also suggest that risk factors such as substance use, poly consumption, engaging in slamsex, and injecting methamphetamine use are associated with an elevated likelihood of experiencing a psychotic break. This association with the risk of psychosis aligns with the results of previously published studies (29, 45).

Associations have been noted between chemsex and an increased risk of suicide. Additionally, there is evidence of an association between chemsex, impulsivity, and distorted body perception. A recent meta-analysis conducted in Sweden (65) reported a suicide attempt rate of 10% in gay men compared to 2.2% in heterosexual men. This suggests that MSM and individuals involved in chemsex may have a higher lifetime risk of suicide attempts compared to the general population (34).

## 5 Strengths and limitations of the study

One of the strengths of our study is that the survey was anonymous, so participants may be especially willing to disclose their chemsex experiences. Other strengths of our study are validated measurement methods, multivariate analysis, and explicit questions about chemsex.

Our findings have certain limitations. Using a cross-sectional study design means that the relationships between variables cannot be interpreted as cause-and-effect. Additionally, the use of non-probability purposive sampling to obtain the sample restricts the generalizability of our results to the broader MSM population. However, the sizable sample size suggests that the results hold validity. Since this is a self-administered survey, the results reflect the respondents’ viewpoints, which may not fully represent the clinical experience. Furthermore, variations in participants’ roles may have influenced their knowledge, experiences, and perceptions of the chemsex phenomenon.

## 6 Conclusion

The study highlights the high prevalence of “chemsex” among men who have sex with men (MSM), increasing the risk of STIs. It underlines the urgent need for preventive strategies and care programs tailored to this population. Chemsex poses unique public health challenges, requiring a comprehensive approach that addresses substance use and sexual health. Individuals involved exhibit high-risk behaviors, along with symptoms of psychopathology and substance dependence. Therefore, identification, education and prevention programs are crucial to mitigate negative consequences. Improving awareness of PrEP as HIV prevention can be effective, as can screening programs for health problems related to drug use, and interventions to increase awareness of risks and provide access to health resources.

## Data availability statement

The raw data supporting the conclusions of this article will be made available by the authors, without undue reservation.

## Ethics statement

The studies involving humans were approved by the Committee of the University of La Rioja with verification code (CSV) (D2R1m2Iu3vLVPdIzGZVVnK0h6N558tCyN). The studies were conducted in accordance with the local legislation and institutional requirements. The participants provided their written informed consent to participate in this study.

## Author contributions

PP-H: Writing – review & editing, Writing – original draft, Methodology, Conceptualization. EB-G: Writing – review & editing, Writing – original draft, Supervision, Conceptualization. AM-S: Writing – original draft, Methodology, Investigation, Data curation, Conceptualization. EC-S: Writing – original draft, Supervision, Software, Methodology, Formal analysis, Conceptualization. VG-C: Writing – original draft, Visualization, Validation, Resources, Project administration, Funding acquisition, Formal analysis. JC-R: Writing – original draft, Methodology, Formal analysis. MC: Writing – original draft, Methodology, Investigation, Conceptualization. PK: Writing – original draft. JM-T: Writing – original draft, Resources, Project administration, Methodology, Investigation. MS-B: Writing – original draft, Project administration, Formal analysis. RV: Writing – original draft, Visualization, Validation, Software, Resources, Project administration, Investigation, Funding acquisition, Formal analysis. RJ-V: Writing – review & editing, Writing – original draft, Supervision, Methodology, Investigation, Funding acquisition, Formal analysis, Conceptualization.
